# Structural and Magnetic Properties of Transition-Metal-Doped Zn _1−*x*_Fe_*x*_O

**DOI:** 10.1186/s11671-016-1332-x

**Published:** 2016-02-29

**Authors:** T. A. Abdel-Baset, Yue-Wen Fang, B. Anis, Chun-Gang Duan, Mahmoud Abdel-Hafiez

**Affiliations:** Faculty of Science, Physics Department, Fayoum University, Fayoum, 63514 Egypt; Key Laboratory of Polar Materials and Devices, Ministry of Education, East China Normal University, Shanghai, 200241 China; Spectroscopy Department, Physics Division, National Research Center, Giza, 12622 Egypt; Center for High Pressure Science and Technology Advanced Research, Shanghai, 201203 China; Institute of Physics, Goethe University Frankfurt, Frankfurt/M, 60438 Germany

**Keywords:** Zinc oxide, Magnetic semiconductors, Ferromagnetism

## Abstract

The ability to produce high-quality single-phase diluted magnetic semiconductors (DMS) is the driving factor to study DMS for spintronics applications. Fe-doped ZnO was synthesized by using a low-temperature co-precipitation technique producing Zn _1−*x*_Fe_*x*_O nanoparticles (*x*= 0, 0.02, 0.04, 0.06, 0.08, and 0.1). Structural, Raman, density functional calculations, and magnetic studies have been carried out in studying the electronic structure and magnetic properties of Fe-doped ZnO. The results show that Fe atoms are substituted by Zn ions successfully. Due to the small ionic radius of Fe ions compared to that of a Zn ions, the crystal size decreases with an increasing dopant concentration. First-principle calculations indicate that the charge state of iron is Fe ^2+^ and Fe ^3+^ with a zinc vacancy or an interstitial oxygen anion, respectively. The calculations predict that the exchange interaction between transition metal ions can switch from the antiferromagnetic coupling into its quasi-degenerate ferromagnetic coupling by external perturbations. This is further supported and explains the observed ferromagnetic bahaviour at magnetic measurements. Magnetic measurements reveal that decreasing particle size increases the ferromagnetism volume fraction. Furthermore, introducing Fe into ZnO induces a strong magnetic moment without any distortion in the geometrical symmetry; it also reveals the ferromagnetic coupling.

## Background

The relation between semiconductors and magnetism has led to the next generation of magnetic semiconductors [1]. Keen interest in spintronic devices consisting of diluted magnetic semiconductors (DMSs) is driven by a possibility to control the magnetism by electric gating. Such devices based on spin and charge degrees of freedom are greatly desired seeing that DMSs are a class of semiconductors where both ferromagnetism and insulating behavior can coexist in a single phase [2]. Particularly in these semiconductor materials, global ferromagnetic order in the entire lattice can be realized by the interplay of spin of the dopant atoms and the carriers [3–8]. ZnO, a widely studied semiconductor with a wide band gap of 3.37 eV, is of growing significance in advanced electronics and spintronics. The exotic properties of ZnO have led [9] and have observed quantum hall effect in a high-mobility two-dimensional electron gas in ZnO-based polar heterostructure [10]. Additionally, ZnO was extensively studied for its transparent conductive oxide aspects, in hope of replacing an indium tin oxide, because it is nontoxic, low cost, and abundant [11]. As in the area of spintronics, technological progress of introducing transition metals like Co, Mn, Fe, Cr, Cu, and Ni has enabled doped ZnO to exhibit excellent magnetic, optical, and electronic properties required for spintronic materials [12–19].

Among these, most researchers have been attracted on the fabrication of Co-Cr-, Ni-, and Mn-doped ZnO systems as well as their structural, optical, and magnetic properties [20–23]. However, the Fe-doped ZnO nanoparticle is still an unsolved problem because some studies show ferromagnetic behavior at room temperature when being prepared by mechanical alloy [24], hydrothermal method [25], a solid-state reaction, and the sol-gel method [26–28]. On the other hand, some reports show that Fe-doped ZnO has an antiferromagnetic nature [29]. The variations in magnetic behavior of Fe-doped ZnO indicate that ferromagnetism of such a system may depend on the methods and conditions used in the preparation.

In transition-metal-doped ZnO, the energy position of the dopant 3*d* states relative to the host conduction and valence bands determining the possibility of long-range ferromagnetism [30]. For Fe-doped ZnO materials, most of the research focuses on the ferromagnetic behavior of Fe-doped ZnO nanoparticles. Although several studies have been proposed, the origin of ferromagnetism in transition meatl-doped ZnO remains very controversial. Similar contradictory results were also observed in Fe-doped ZnO nanomaterials [31–33]. Sharma et al. [34] demonstrated that 0.01 Fe-doped ZnO samples show a diamagnetic character, while ferromagnetic nature is observed for 0.02 and 0.03 Fe-doped samples, and the higher doping of Fe. However, to the best of our knowledge, no straight forward procedure has been reported yet for fabricating reproducible and stable transition-metal-doped ZnO of high quality.

In this work, we concentrate on Fe-doped ZnO bulk samples. We examine five samples grown with a varied Fe content from 0.02 to 0.1. The weight percents were calculated from the weight of Fe_2_O_3_ vs. ZnO in the starting material. The idea of additional Fe doping in Zn _1−*x*_Fe_*x*_O was highly successful and led us to a ferromagnetic DMS. We present a low-cost and suitable method for synthesizing Fe-doped ZnO nanoparticles with a semispherical shape, without using a surfactant. X-ray diffraction confirms that the samples have a single-phase wurtzite structure where the crystal size decreases with an increasing dopant concentration. Raman studies show that the local symmetry in the Zn _1−*x*_Fe_*x*_O nanocrystals is different from that of pure ZnO. From these results, we have discussed the origin of the ferromagnetism of Zn _1−*x*_Fe_*x*_O. Introducing Fe into ZnO induces a strong magnetic moment without any distortion in the geometrical symmetry; it also reveals the ferromagnetic coupling. The exchange interaction between transition metal ions can switch from the antiferromagnetic coupling into its quasi-degenerate ferromagnetic coupling by external perturbations, which is obtained by first-principle calculations.

## Methods

The material synthesis is one of the key features for the development and realization of semiconductor based spintronic applications. All preparation steps like weighing, mixing, grinding, and storage were carried out in an Ar-filled glove box; the O_2_ and H_2_O level is less than 0.1 ppm. The preparation of Zn _1−*x*_Fe_*x*_O in a nanoparticle form is achieved by using the co-precipitation technique. The following procedure was adopted.

ZnCl_2_ and NaOH solution were prepared separately and then mixed together. The solution was maintained at room temperature stirring for 2 h and heating of Zn(OH)_2_ at 70 °C for 24 h for drying. The dryed ingots were heated at 400 °C for 4 h; after that time period, the powder was left to cool down slowly to room temperature to get pure ZnO. To prepare mixed oxide dilute magnetic semiconductors, mixed solutions of ZnCl_2_ and FeCl_2_ at the desired ratio were prepared; then NaOH solution was added slowly to the mixed solution, and the process described above is repeated to obtain Zn _1−*x*_Fe_*x*_O nanoparticles.

The X-ray powder diffraction data were collected at room temperature using a Huber G670 Guinier imaging plate diffractometer with Co K *α*-radiation and a Ge-111 monochromator. Scanning electron microscopy (SEM) images of the surface and cross section of films were taken with a Leo Gemini 982 microscope. Raman studies were obtained from their vibration modes in wave number range of 50–1500 cm ^−1^ using a Jobin-Yvon Raman spectrophotometer with the incident laser power of 40 mW. The magnetization measurements were performed by using a superconducting quantum interference device magnetometer (MPMS-XL5) from Quantum Design.

First-principle calculations within the framework of density functional theory (DFT) are performed by Vienna Ab initio Simulation Package (VASP) [35, 36]. A kinetic energy cutoff is set to be 550 eV for the plane wave basis and the Brillouin zone integration is sampled by using a 8×8×6 Monkhorst-Pack grid. The exchange-correlation functional is treated in the form of Perdew-Burke-Ernzerhof (PBE) [37]. In the relaxations, the lattice parameters are fixed on the experimental lattice constants of ZnO, and the atomic positions are fully relaxed until the Hellmann-Feynman forces on each atom are less than 1 meV/Å and the total energies are converged to within 10 ^−3^ meV/atom. To obtain highly accurate energy and density of states (DOS), we use the linear tetrahedron method with Blöchl corrections throughout the whole computations. In addition, we introduce an effective *Hubbard* parameter *U*= 4 eV to better describe the onsite Coulomb interactions of *d* electrons for Fe.

## Results and discussion

**Fig. 1:**
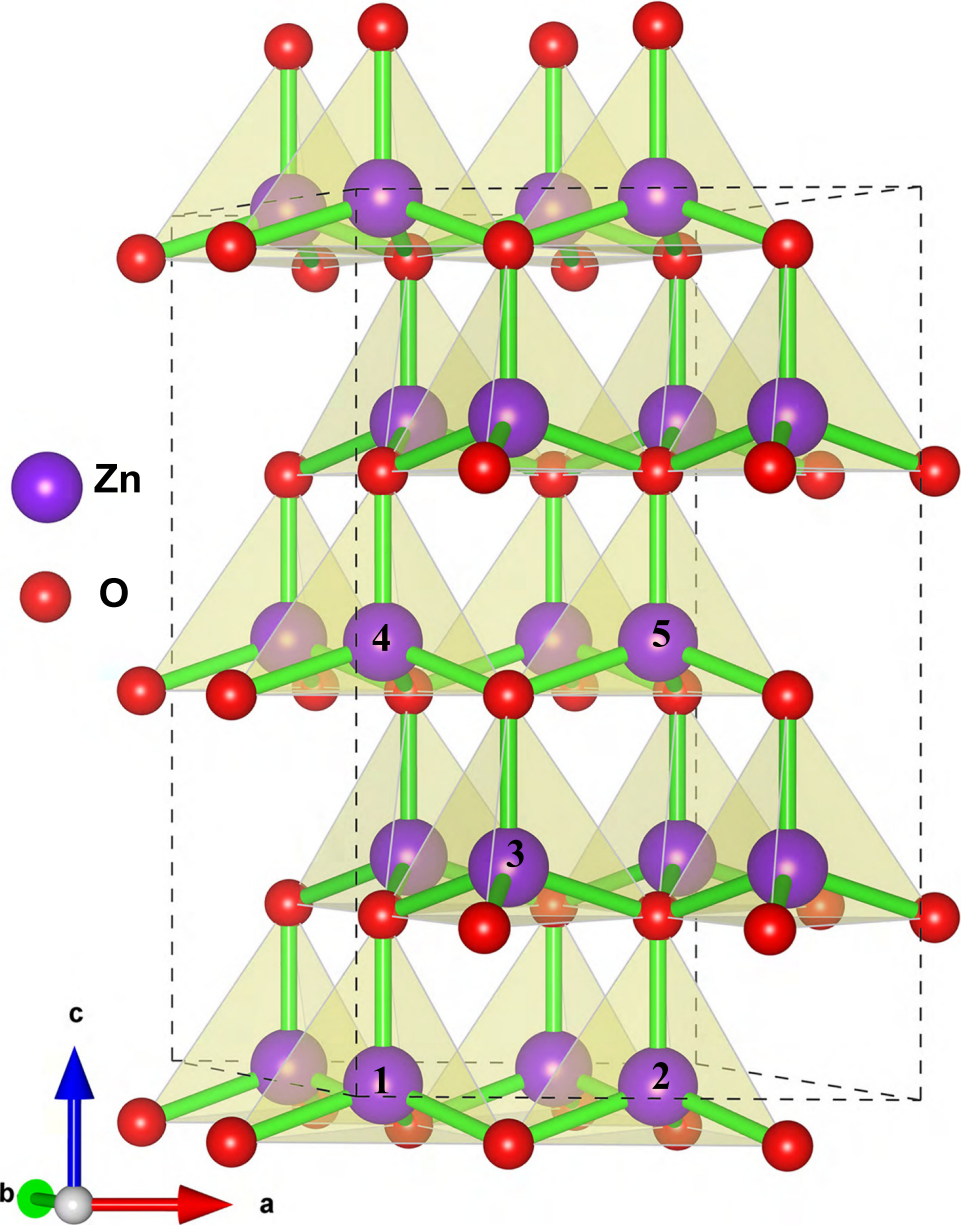
Schematic diagram of 2 × 2 × 2 ZnO supercell with a hexagonal wurtzite structure. The *numbers* on the spheres indicate the atomic positions substituted by Fe cations in subsequent first-principle calculations. The atom labeled *1* is the reference position

The most common lattice of ZnO is recognized as a hexagonal wurtzite structure as shown in Fig. 1. The numbers in Fig. 1 on the spheres illustrate the atomic positions substituted by Fe cations in subsequent first-principle calculations. The atom labeled 1 is the reference position. In our study, the structure of pure ZnO and Zn _1−*x*_Fe_*x*_O are both carefully characterized.

**Fig. 2:**
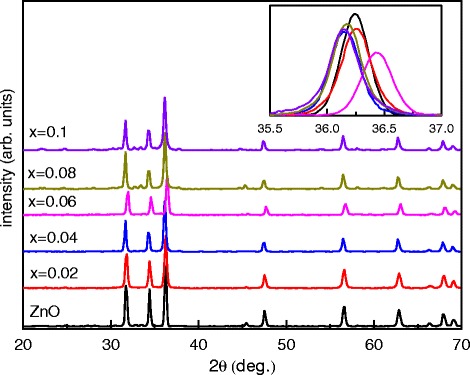
XRD patterns of the pure ZnO and the Fe-doped ZnO. The *inset* shows the magnified of the (101) plane showing the peak shift

Figure 2 shows the X-ray diffraction pattern of the Zn _1−*x*_Fe_*x*_O nanostructure compared to the pure ZnO nanostructure. The XRD pattern suggests that the pure ZnO exhibits a hexagonal wurtzite structure (belonging to the C46v space group (P63mc). It is indext and uses a standard JCPDS file for ZnO (JCPDS 36-1451)) with a preferred (101) orientation. The diffraction peaks corresponding to (002) and (100) planes of ZnO a hexagonal phase were also observed but with a different intensity ratio. One can observe that the hexagonal wurtzite planes show a small shift with an increasing Fe content except the sample doped with 0.06. It is well known that the Fe ions have two oxidation states Fe ^+2^ and Fe ^+3^, where the ionic radius of Fe ^+2^ is 0.78Å which is bigger than that of Zn ^+2^ (0.74Å) whereas that of Fe ^+3^ is smaller by about 10 % [38]. Therefore, the hexagonal wurtzite structure will not strongly change with replacing Zn ^+2^ with Fe ^+2^ ions. When the Zn ^+2^ ions are replaced with Fe ^+3^ ions, the oxygen ions would be drawn to the Fe ^+3^ ions to keep the balance of charge [39, 40]. Based on the above discussions, we can conclude that the large shift of the crystalline peaks for the *x*=0.06 sample could be attributed to the replacement of the Zn ^+2^ by Fe ^+3^ ions. This variation was previously observed and attributed to the different ionic radii of Fe ions substituted in the ZnO lattice [41].

Table 1 depicts the lattice constants for the Zn _1−*x*_Fe_*x*_O samples compared to those of a pure ZnO. The lattice parameters *a* and *c* do not show any significant change compared to those of the pure ZnO sample except the *x*= 0.06 sample. This change could be attributed to the substitution of the Zn ^+2^ with Fe ^+3^ ions in the hexagonal structure. The lattice parameters *a* and *c* are calculated by using the relations [42]

(1) $$ \frac{1}{a^{2}}= \frac{4}{3a^{2}}\left[h^{2} + hk + k^{2}\right]+\frac{l^{2}}{c^{2}}.  $$

**Table 1:** The calculated values of the lattice constants and the crystal size for all samples investigated for Zn _1−*x*_Fe_*x*_O at (101)

*x*	*a*	*c*	*c*/*a*	*D* (nm)
0	3.257	5.203	1.6	31.4
0.02	3.245	5.203	1.603	28.5
0.04	3.269	3.220	1.6	29.6
0.06	3.233	3.185	1.603	28.5
0.08	3.257	3.220	1.602	29.8
0.1	3.257	3.220	1.602	29.2

The crystal size (*D*) was calculated by using Debye-Scherrer’s equation [43, 44]: The *D*=$\frac {0.94\lambda }{\beta \cos (\Theta)}$, where *D* is the particle size, *λ* is the wavelength of radiation, *β* is the full width at half maxima (FWHM), and *Θ* is the Bragg angle.

The calculated values are listed in Table 1. From the table, one can observe that with the increasing concentration of the iron ion, the particle size decreases. This decrease in the particle size was previously observed and attributed to the decrease in the nucleation and subsequently growth rate due to the difference between the Fe ions (Fe ^+2^ and Fe ^+3^) and Z ^+2^ ions [38, 45].

**Fig. 3:**
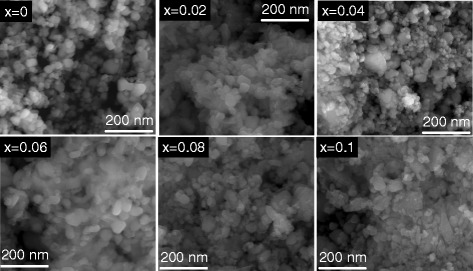
SEM images for Zn _1−*x*_Fe_*x*_O samples, for Fe concentrations 0, 0.02 0.04, 0.06, 0.08, and 0.1, respectively

The microstructure of the Zn _1−*x*_Fe_*x*_O nanoparticles was investigated by SEM, as shown in Fig. 3. It clearly proofs that the structures of the investigated particles are crystals in form. In addition, the particles show a narrow size distribution due to their magnetic attraction exhibiting a partially sintered microstructure. It should be noticed that the agglomeration of particles were related to many factors such as the shape factor, surface area, porosity, and density. It is worth mentioning that the most colloidal particles are electrically charged, e.g., most metal oxides have a surface layer of the metal hydroxide which is amphoteric and can become either positively or negatively charged. From Fig. 3, one can observe that a pure ZnO sample has a spherical shape (with 30 nm average particle size) where the doped samples show (with 28 nm average particle size) compared to the pure sample.

**Fig. 4:**
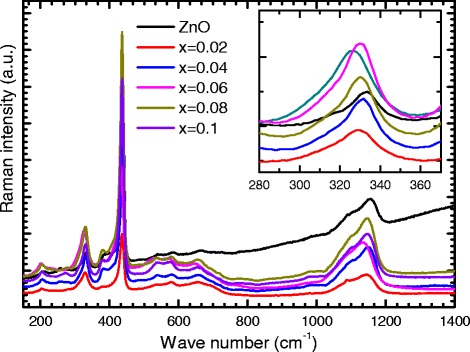
The main frame represents the Raman spectra of Zn_1−*x*_Fe_*x*_O samples. The *inset* shows the magnified of the A1 (TO) mode showing the peak shift

In order to study the modified lattice dynamics, we performed Raman studies for all samples. Raman spectroscopy is a non-invasive technique and the elementary excitations detected by Raman scattering are phonons. Therefore, Raman can provide us with many information on the structural properties or crystalline quality. Figure 4 shows the Raman spectra for the pure ZnO nanocystals. In hexagonal structures with a C46v symmetry like ZnO, six sets of phonon normal modes at the center of the Brillouin zone (*Γ* point) are optically active modes [46–48]. The phonons of the Wurtzite ZnO belong to the following irreducible representation:

(2) $$ \Gamma = A_{1} + E_{1} + 2B_{1} + 2E_{2},  $$

where *A*_1_ and *E*_1_ modes are two polar branches and split into the transverse optical (TO) and longitudinal optical (LO) phonons, resulting from beating along the c-axis, with different frequencies due to the macroscopic electric fields associated with LO phonons. For lattice vibrations with *A*_1_ and *E*_1_ symmetry, the atoms move parallel and perpendicular to the c-axis, respectively. The two nonpolar *E*_2_ branches are Raman active only, and the *B*_1_ modes are generally inactive in Raman spectra and are called silent modes [49].

The Raman spectrum of ZnO nanoparticles consists of six peaks located at about 200, 332, 377, 437, 600, and 1144 cm ^−1^. The 200 peak is due to *E*_2_ (LO) possibly related to the doping of the zinc oxide layer and to the charge carrier concentration; the 332-cm ^−1^ peak is due to an *A*_1_ (TO) mode which shows a second-order Raman processes (multiple-phonon processes). The 377-cm ^−1^ peak is due to an *E*_1_ (TO) mode. Also, the sharpest and the strongest peak at 437 cm ^−1^ can be assigned *E*_2_ (high) due to the high-frequency branch of *E*_2_ mode of ZnO, which is the strongest and characteristic mode of wurtzite crystal structure [50–55]. Raman peak corresponding to the high-energy range observed between ≈1030 and 1190 cm ^−1^ is assigned to the *E*_2_ (LO) second-order polar mode [56].

The Raman spectra of Zn _1−*x*_Fe_*x*_O nanocrystals are shown in Fig. 4. It is clear from the figure that all the ZnO peaks are also observed in Fe-doped samples. As the Fe content increases, the ZnO peaks shift toward lower frequencies expect the *x* = 0.06 sample which shows a relatively large shift (see the inset of Fig. 4). This means that the local symmetry in the Zn _1−*x*_Fe_*x*_O nanocrystals is different from that of the pure ZnO, but the crystal structure is the same. The relatively large shift in the *x*= 0.06 sample could be attributed to the bonds softening in the Zn-O bond as a result of substitution of Zn ^+2^ with Fe ^+3^. The Fe ^3+^ ions have relatively large electro-negativity than the Fe ^2+^ and Zn ^2+^; this could reduce the strength of the Zn-O bond, hence leading to shift the peak to lower energy [57, 58].

**Fig. 5:**
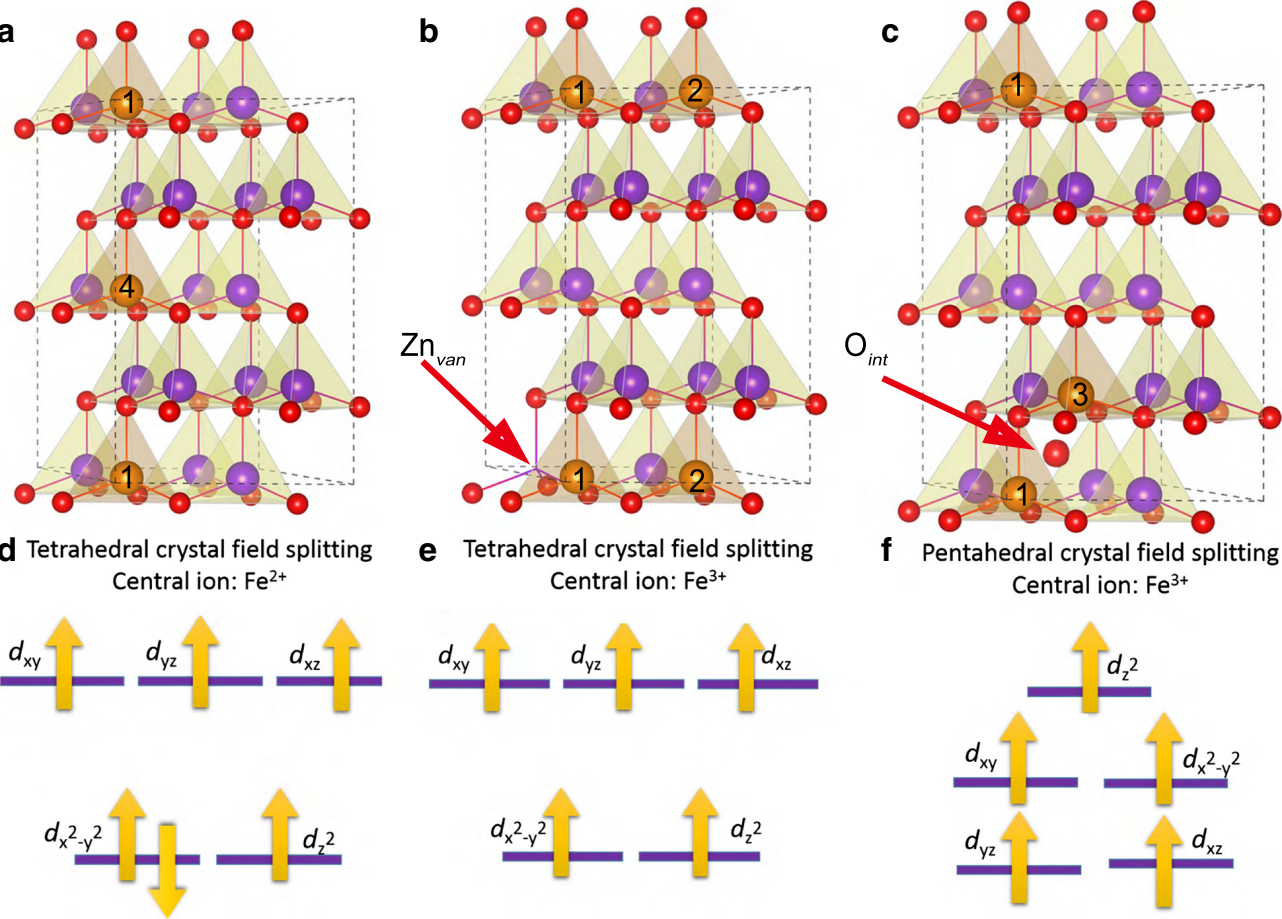
Schematic diagram of the most stable structures (*upper panel*) and their corresponding crystal field splitting (*bottom panel*) in our calculations for Zn _1−*x*_Fe_*x*_O, Zn _1−*x*_Fe_*x*_O with Zn _van_, and Zn _1−*x*_Fe_*x*_O with O _int_, respectively (**a**–**f**)

In order to explore the magnetic and electronic properties of Fe-doped ZnO, we adopt 2×2×2 Zn _1−*x*_Fe_*x*_O superlattices to perform first-principle calculations. The models with substitutional dopants, zinc vacancy (Zn _van_) and interpolated oxygen (O _int_) were all considered. Figure 5a–c shows the most stable structures obtained in total energy calculations. We also calculate the energy differences (meV/Fe-cation) between the ferromagnetic state and the antiferromagnetic state for the above stable cases. The energy difference indicates the interactomic exchange interaction, and it is proportional to phase transition temperature within the framework of mean-field theory.

In our calculations, using the charge analyses, the respective oxidation states of Fe atoms are Fe ^2+^ in the case of Fig. 5a and Fe ^3+^ in Fig. 5b, c; it is unexpected that all these stable cases energetically favor antiferromagnetic states. Due to that all the exchange interactions in these cases are mediated by the oxygen anions, we can employ the semi-empirical Goodenough-Kanamori-Anderson rules [59, 60] to analyze the superexchange for each case qualitatively, in combination with the first-principle calculations. In the case of Fig. 5a with Fe ^2+^ ions, Fe ^2+^-Fe ^2+^ coupling favors antiferromagnetic interaction can be understood by the 180° Fe-O-Fe superexchange contributed by the virtual electron hopping between the half-filled *t*_2*g*_ orbitals (see Fig. 5d). In the case of Zn _1−*x*_Fe_*x*_O with Zn _van_ in Fig. 5b, we find that the angle of Fe ^3+^-O-Fe ^3+^ is 116.1°, as suggested by the Goodenough-Kanamori-Anderson rules for transition metal oxides [60], a 90° Fe ^3+^-O-Fe ^3+^ angle usually leads to ferromagnetic coupling while a 180° Fe ^3+^-O-Fe ^3+^ angle favors antiferromagnetic coupling. Consequently, in this situation with a 116.1° Fe ^3+^-O-Fe ^3+^ angle, there is a competition between ferromagnetic and antiferromagnetic superexchange. As calculated in our first-principle calculations, Zn _1−*x*_Fe_*x*_O with Zn _van_ is finally compromised into an antiferromagnetic state.

On the other hand, one can further find from Fig. 5b that the sites of two Fe cations adopt a close configuration in which two Fe atoms locate at the same *xy* plane. This indicates that introductions of Zn _van_ might tend to a cluster together the Fe ions during growth, rather than evenly distribute them through the entire lattice, such a dynamical behavior can inhibit the formation of global magnetism owning to the localization of magnetic states. For the last case incorporated of an O _int_ in Fig. 5c, the crystal field splitting of magnetic ion Fe _3+_ is very different from those in the previous two cases, as seen in Fig. 5f, where an interpolated oxygen atom and its neighboring oxygen atoms form a pentahedral ligand field and lift the energy level of $d_{z^{2}}$. The virtual electron hopping between the two half-filled $d_{z^{2}}$ orbitals gives rise to the antiferromagnetic coupling of Fe ^3+^-Fe ^3+^. In addition, we obtain from our total energy calculations that ferromagnetic states for the structures in Fig. 5a, c is only 1.8 and 2.0 meV energetically disfavored, respectively. The scale of these energy differences is quite small in first-principle calculations; thus, the antiferromagnetic and the magnetic state in these two cases can be thought as quasi-degenerate states. It can be predicted that the ferromagnetism cloud be brought by a small applied external perturbations.

**Fig. 6:**
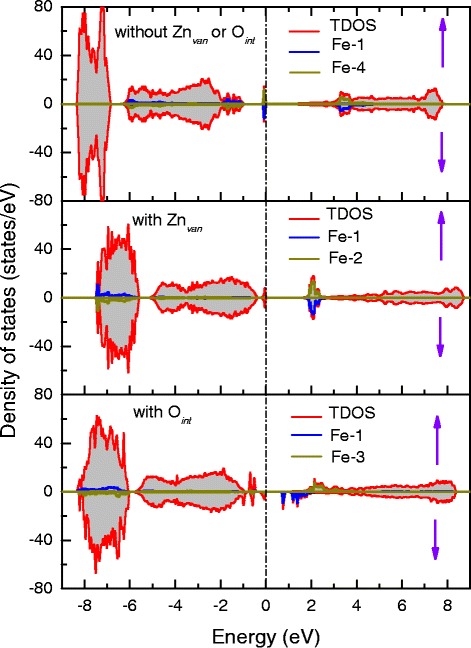
Density of states of Zn _1−*x*_Fe_*x*_O (*upper* panel), Zn _1−*x*_Fe_*x*_O with Zn _*van*_ (*middle* panel) and Zn _1−*x*_Fe_*x*_O with O _*int*_ (*bottom* panel), respectively. *Red* line, as well as the light grey area denotes TDOS; *blue* and *dark yellow* lines denote PDOS of Fe atoms. The Fermi energy is set to zero. Upwards and downwards arrows denote the majority and minority spin channels, respectively

The calculated total density of states (TDOS) and the projected density of states (PDOS) of Fe atoms are presented in Fig. 6. Due to that, each model which is used in our calculations contains two Fe atoms. We have plotted the PDOS separately, with the reference Fe atom shown by a blue line and the other one by a dark yellow line. Correspondingly to the above discussions, DOS in all cases which remain insulted shows antiferromagnetic features, indicating all structures remain insulating with the existence of Zn _van_ or O _int_. Compared to the TDOS of Zn _1−*x*_Fe_*x*_O, one can observe that the Fermi level shifts down and the energy gap narrows in Zn _1−*x*_Fe_*x*_O with Zn _van_ or O _int_. Furthermore, it can be seen from the PDOS that the exchange splitting energy is much larger than the crystal field splitting energy; it opens the energy gap in each case. The Fe-3*d* manifold is split by a large exchange splitting into distinct states in two spin channels, each of which in turn is split by the smaller tetrahedral crystal field or pentahedral crystal field. In the energy range from −8 to −6.5 eV, the Fe-3*d* states are strongly hybridized with O-2*p* states in Zn _1−*x*_Fe_*x*_O with Zn _van_ or O _int_; in contrast, they vanish in Zn _1−*x*_Fe_*x*_O without additional defects, and we think that these states tightly related to covalent bonding might contribute to the Fe-O-Fe superexchange.

**Fig. 7:**
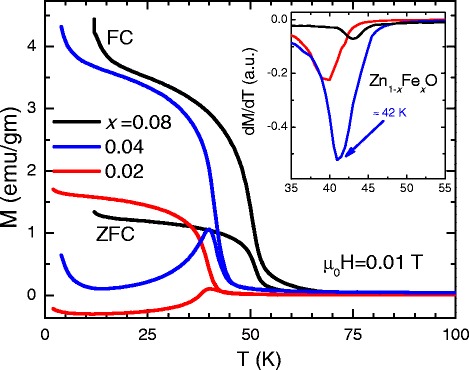
Temperature dependence of magnetization measured under a field of 100 Oe with ZFC and FC histories for *x* = 0.02, 0.04, and 0.08. The inset exhibits the derivative plot of magnetization, dM/dT, to elucidate the transition temperature

**Fig. 8:**
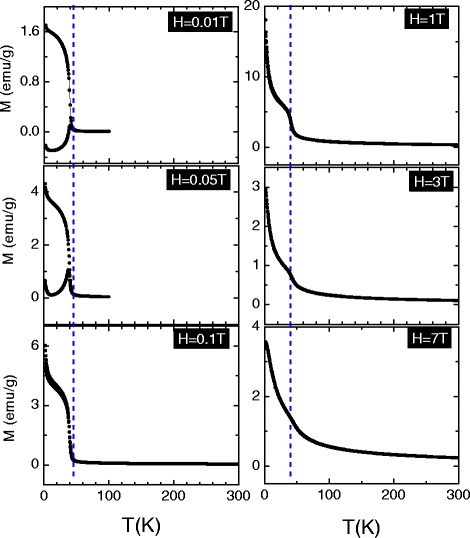
The temperature dependence of magnetization measured under various applied fields with ZFC and FC measurements for *x* = 0.02. Clearly, when applying upon a high magnetic field the ZFC curve, it overlaps with the FC curve

Motivated by the predictions of mergence of magnetism in first-principle calculations, we perform the magnetic characterization of our samples. The temperature dependencies of magnetization zero-field cooling (ZFC) and field cooling (FC) are shown in Fig. 7 for 2, 4, and 8 % Fe-doped the ZnO samples, respectively. The ZFC measurements were performed by warming the sample, after prior cooling it to a low temperature in the absence of a magnetic field, followed by the application of the field of a given strength. The FC data were also recorded on warming, after previous cooling of the sample in a magnetic field. A sharp ferromagnetic transition is seen at *T*_*c*_ for all investigated samples, which the *T*_*c*_ is accurately estimated from the derivative plot of magnetization data (inset of Fig. 7). Obviously, one can see that Fe doping enhances the ferromagnetism of Zn _1−*x*_Fe_*x*_O with *T*_*c*_ increasing from 39 K for the 2 % to 44 K for the 8 %. For all study samples, the ferromagnetic transition and the magnetic susceptibility decreases sharply and continuously at low temperature as seen clearly in low-field plots. As shown in Fig. 8, this decreasing disappears upon applying high magnetic field and the ZFC curve overlaps with the FC curve. This downturn can be attributed to the antiphase ferromagnetic domains.

For better understanding the nature of the ferromagnetic transitions, the temperature dependence of magnetization at different applied fields below and above the critical temperature has been measured. Figure 8 presents ZFC and FC data for six values of the applied magnetic fields only. At high temperatures, the ZFC and FC magnetizations match each other and gradually increase as temperature decreases, probably due to local clustering of the spins [61] or to ferromagnetic domain growth [62]. The ZFC and FC magnetization start to differ below a certain field-dependent temperature *T*_*f*_(H), which is a phenomenon that can be interpreted in terms of a spin-glass transition or by freezing domain walls motion [63].

**Fig. 9:**
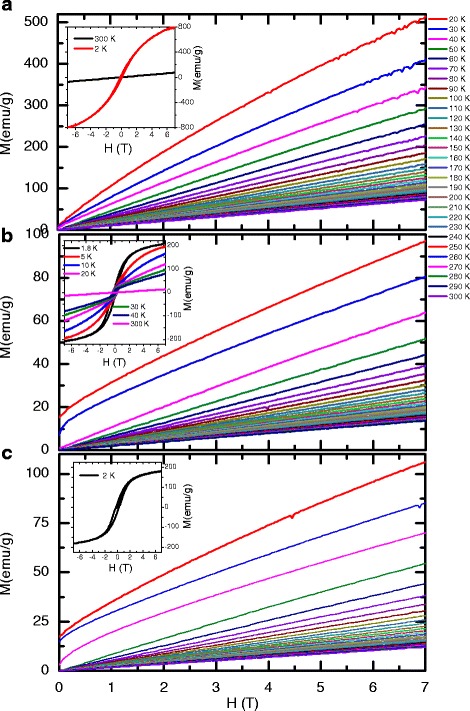
**a**–**c** Magnetization vs. field isotherms from 20 to 300 K for *x*= 0.02, 0.04, and 0.08, respectively. The *insets* illustrate the isothermal magnetization plots at 2 and 300 K up to +7 and −7 T suggesting the ferromagnetic signature at our investigated systems

Further signatures of the ferromagnetic behavior is observed from the magnetic hysteresis loop at 2 K (inset of Fig. 9). Although the *T*_*c*_ is increasing by introducing Fe to the ZnO system, only a signature of single-phase ferromagnetic ordering is evident in the temperature dependence of *x*=0.02, 0.04, and 0.08 (Fig. 9). It is well known that the pure ZnO nanoparticles are paramagnetic materials. Fe-doped ZnO exhibits a clearly ferromagnetic hysteresis loop (see the insets of Fig. 9). As reported by Xing et al., the appearance of ferromagnetism in transition metal-doped ZnO might be due to the increase of the number of defects and oxygen vacancies [64]. On the other hand, theoretically, Chu et al. prove that ferromagnetism could be induced by the exchange interaction between transition metal ions and O ion spin moments [65].

## Conclusions

Summarizing, we have grown a Zn _1−*x*_Fe_*x*_O system and characterized its structural, electronic, and magnetic properties through XRD, SEM, electronic, magnetization, and DFT. X-ray diffraction confirms that the samples have a single-phase wurtzite structure which the main crystal size decreases with an increasing dopant concentration. This decrease occurs due to the small ionic radius of Fe ions in comparison to Zn ions. To ensure the superstructure electrically neutral, we replaced Fe ^2+^ ions by Zn ^2+^ ions. Additionally, Fe ^3+^ ions are introduced either by a Zn vacancy or an interpolated O. Magnetic measurements reveal that when the particle sizes decrease, then the FM volume fraction decreases. The ferromagnetism is enhanced caused by Fe doping to the ZnO.

This work was supported by the 973 Programs (No. 2014CB921104, 2013CB922301) and the NSF of China (No. 51572085). The work in Germany is supported by the program MO 3014/1-1 of the DFG. The computations are conducted at ECNU computing center and Chinese Tianhe-1A system at the National Supercomputer Center.

## References

[1] Dietl T (2010). A ten-year perspective on dilute magnetic semiconductors and oxides. Nat Mater.

[2] Sato K, Katayama-Yoshida H (2000). Material design for transparent ferromagnets with ZnO-based magnetic semiconductors. Jpn J Appl Phys.

[3] Zunger A, Lany A, Raebiger H (2010). Trend: the quest for dilute ferromagnetism in semiconductors: guides and misguides by theory. Physics.

[4] Wang Y, Zhan Y, Pang M (2012). Electronic structures and magnetism of diluted magnetic semiconductors Sn _1−*x*_ Gd _*x*_ Te: A density functional theory study. J Appl Phys.

[5] Dietl T, Ohno H, Matsukura F, Cibert J, Ferrand D (2000). Zener model description of ferromagnetism in zinc-blende magnetic semiconductors. Science.

[6] Hong NH, Sakai J, Huong NT, Poirot N, Ruyter A (2005). Role of defects in tuning ferromagnetism in diluted magnetic oxide thin films. Phys Rev B.

[7] Sato K, Yoshida HK (2000). Material Design for Transparent Ferromagnets with ZnO-Based Magnetic Semiconductors. J Appl Phys.

[8] Sluiter MHF, Kawazoe Y, Sharma P, Inoue A, Raju AR, Rout C, Waghmare UV (2005). First Principles Based Design and Experimental Evidence for a ZnO-Based Ferromagnet at Room Temperature. Phys Rev Lett.

[9] Tsukazaki A, Ohtomo A, Onuma T, Ohtani M, Makino T, Sumiya M, Ohtani K, Chichibu SF, Fuke S, Segawa Y, Ohno H, Koinuma H, Kawasaki M (2005). Repeated temperature modulation epitaxy for p-type doping and light-emitting diode based on ZnO. Nat Mater.

[10] Tsukazaki A, Ohtomo A, Kita T, Ohno Y, Ohno H, Kawasaki M (2007). Quantum Hall Effect in Polar Oxide Heterostructures. Science.

[11] T-Thienprasert J, Rujirawat S, Klysubun W, Duenow J, Coutts TJ, Zhang SB, Look DC, Limpijumnong S (2013). Compensation in Al-Doped ZnO by Al-Related Acceptor Complexes: Synchrotron X-Ray Absorption Spectroscopy and Theory. Phys Rev Lett.

[12] Li F, Yuan Y, Luo J, Qin Q, Wu J, Li Z, Huang X (2010). Synthesis and characterization of ZnO-Ag core-shell nanocomposites with uniform thin silver layers. Appl Surf Sci.

[13] Wang Q, Geng B, Wang S (2009). ZnO/Au Hybrid Nanoarchitectures: Wet-Chemical Synthesis and Structurally Enhanced Photocatalytic Performance. Environ Sci Technol.

[14] Ungureanu M, Schmidt H, Wenckstern HV, Hochmuth H, Lorenz M, Grundmann M, Fecioru-Morariu M, Guntherodt G (2007). A comparison between ZnO films doped with 3d and 4f magnetic ions. Thin Solid Films.

[15] Zhou Y, Lu SX, Xu WG (2009). Photocatalytic activity of Nd-doped ZnO for the degradation of C.I. Reactive Blue 4 in aqueous suspension. Environ Prog Sust Energ.

[16] Lawes G, Risbud AS, Ramirez AP, Seshadri R (2005). Absence of ferromagnetism in Co and Mn substituted polycrystalline ZnO. Phys Rev B.

[17] Risbud AS, Spaldin NA, Chen ZQ, Stemmer S, Seshadri R (2003). Magnetism in polycrystalline cobalt-substituted zinc oxide. Phys Rev B.

[18] Rao CNR, Deepak FL (2005). Absence of ferromagnetism in Mn- and Co-doped ZnO. J Mater Chem.

[19] Thota S, Dutta T, Kumar J (2006). On the sol-gel synthesis and thermal, structural, and magnetic studies of transition metal (Ni, Co, Mn) containing ZnO powders. J Phys Condens Matter.

[20] Liu M, Kitai AH, Mascher P (1992). Point defects and luminescence centres in zinc oxide and zinc oxide doped with manganese. J Lumin.

[21] Pearton SJ, Abernathy CR, Overberg ME, Thaler GT, Norton DP, Theodoropoulou N, Hebard AF, Park YD (2003). Wide band gap ferromagnetic semiconductors and oxides. J Appl Phys.

[22] Ueda K, Tabat H, Kawai T (2001). Magnetic and electric properties of transition-metal-doped ZnO films. Appl Phys Lett.

[23] Liu Y, Yang J, Guan Q, Yang L, Zhang Y, Wang Y, Feng B, Cao J, Liu X, Yang Y, Wei M (2009). Effects of Cr-doping on the optical and magnetic properties in ZnO nanoparticles prepared by sol-gel method. J Alloys Compd.

[24] Lin Y, Jiang D, Lin F, Shi W, Ma X (2007). Fe-doped ZnO magnetic semiconductor by mechanical alloying. J Alloys Compd.

[25] Wu X, Wei Z, Zhang L, Wang X, Yang H, Jiang J (2014) Optical and Magnetic Properties of Fe Doped ZnO Nanoparticles Obtained by Hydrothermal Synthesis. J Nanomater2014: Article ID 792102.

[26] Beltr JJ, Barrero CA, Punnoose A (2015). Understanding the role of iron in the magnetism of Fe doped ZnO nanoparticles. Phys Chem Chem Phys.

[27] Ahn GY, Park SI, Kim SJ, Lee BW, Kim CS (2005). Preparation of Fe-doped ZnO ferromagnetic semiconductor by sol-gel method with hydrogen treatment. IEEE Trans Magn.

[28] Ahn GY, Park SI, Kim CS (2006). Enhanced ferromagnetic properties of diluted Fe doped ZnO with hydrogen treatment. The 6th Int Symp on Physics of Magnetic Materials. J Magn Magn Mater.

[29] Yoon SW, Cho SB, We SC, Yoon S, Suh BJ, Song HK, Shin J (2003). Magnetic properties of ZnO-based diluted magnetic semiconductors. J Appl Phys Lett.

[30] Sarsari IA, Pemmaraju CD, Salamati H, Sanvito S (2013). Many-body quasiparticle spectrum of Co-doped ZnO: A GW perspective. Phys Rev B.

[31] Karmakar D, Mandal SK, Kadam RM, Paulose PL, Rajarajan AK, Nath TK, Das AK, Dasgupta I, Das GP (2007). Ferromagnetism in Fe-doped ZnO nanocrystals: Experiment and theory. Phys Rev B.

[32] Kataoka T, Kobayashi M, Sakamoto Y, Song GS, Fujimori A, Chang FH, Lin HJ, Huang DJ, Chen CT, Ohkochi T, Takeda Y, Okane T, Saitoh Y, Yamagami H, Tanaka A, Mandal SK, Nath TK, Karmakar D, Dasgupta I (2010). Electronic structure and magnetism of the diluted magnetic semiconductor Fe-doped ZnO nanoparticles. J Appl Phys.

[33] Gu H, Jiang Y, Yan M (2012). Defect-induced room temperature ferromagnetism in Fe and Na co-doped ZnO nanoparticles. J Alloys Comp.

[34] Sharma PK, Dutta RK, Pandey AC, Layek S, Verma HC (2009). Effect of iron doping concentration on magnetic properties of ZnO nanoparticles. J Magn Mater.

[35] Kresse G, Hafner J (1993). Ab initio molecular dynamics for liquid metals. Phys Rev B.

[36] Kresse G, Hafner J (1994). Ab initio molecular-dynamics simulation of the liquid-metal-amorphous-semiconductor transition in germanium. Phys Rev B.

[37] Perdew JP, Burke K, Ernzerhof M (1996). Generalized gradient approximation made simple. Phys Rev Lett.

[38] Jin Z, Fukumura T, Kawasaki M, Ando K, Saito H, Sekiguchi T, Yoo YZ, Murakami M, Matsumoto Y, Hasegawa T, Koinuma H (2001). High throughput fabrication of transition-metal-doped epitaxial ZnO thin films: A series of oxide-diluted magnetic semiconductors and their properties. Appl Phys Lett.

[39] Cheng W, Ma X (2009). Structural, optical and magnetic properties of Fe-doped ZnO. J Phys Conf Ser.

[40] Zhang Y, Wu L, Li H, Xu J, Han L, Wang B, Tuo Z, Xie E (2009). Influence of Fe doping on the optical property of ZnO films. J Alloys Compd.

[41] Karamat S, Rawat RS, Lee P, Tan TL, Ramanujan RV (2014). Prog Nat Sci Mater Int.

[42] Karyaoui M, Mhamdi A, Kaouach H, Labidi A, Boukhachem A, Boubaker K, Amlouk M, Chtourou R (2015). Some physical investigations on silver-doped ZnO sprayed thin films. Mater Sci Semicond Process.

[43] Cullity BD (1978). Elements of X-ray diffraction.

[44] Patterson L (1939). The Diffraction of X-Rays by Small Crystalline Particles. Phys Rev.

[45] Mishra AK, Das D (2010). Investigation on Fe-doped ZnO nanostructures prepared by a chemical route. Mater Sci Eng B.

[46] Singh S, Srinivasa RS, Major SS (2007). Effect of substrate temperature on the structure and optical properties of ZnO thin films deposited by reactive rf magnetron sputtering. Thin Solid Films.

[47] Song D (2008). Effects of rf power on surface-morphological, structural and electrical properties of aluminium-doped zinc oxide films by magnetron sputtering. Appl Surf Sci.

[48] Morko H, Ozgur U (2009) Fundamentals, materials and device technology. Wiley VCH. ISBN: 978-3-527-40813-9.

[49] Wang JB, Huang GJ, Zhong XL, Sun LZ, Zho YC, Liu EH (2006). Raman scattering and high temperature ferromagnetism of Mn-doped ZnO nanoparticles. Appl Phys Lett.

[50] Srinivas K, Rao SM, Reddy PV (2011). Preparation and properties of Zn 0.9 Ni 0.1 O diluted magnetic semiconductor nanoparticles. J Nanopart Res.

[51] Duan LB, Zhao XR, Liu JM, Wang T, Rao GH (2010). Room-temperature ferromagnetism in lightly Cr-doped ZnO nanoparticles. Appl Phys A.

[52] Aljawfi RN, Rahman F, Batoo KM (2013). Surface defect mediated magnetic interactions and ferromagnetism in Cr/Co Co-doped ZnO nanoparticles. J Magn Magn Mater.

[53] Kaushik A, Dalela B, Rathore R, Vats VS, choudhary BL, Alvi PA, Kujar S, Ddalela S (2013). Influence of Co doping on the structural, optical and magnetic properties of ZnO nanocrystals. J Alloys Compd.

[54] Sahare PD, Kumar V (2013). Optical and Magnetic Properties of Cu-Doped ZnO Nanoparticles. Int J Innov Tech Exploring Engin.

[55] Singh S, Dey P, Roy JN, Mandal SK (2014). Enhancement of dielectric constant in transition metal doped ZnO. A Phys Lett.

[56] Pandiyarajan T, Udayabhaskar R, Karthikeyan B (2013). Microstructure and enhanced exciton-phonon coupling in Fe doped ZnO nanoparticles. Spectrochim Acta A.

[57] Parra-Palomino A, Perales-Perez O, Singhal R, Tomar M, wang J, Voyles PM (2008). Structural, optical, and magnetic characterization of monodisperse Fe-doped ZnO nanocrystals. J Appl Phys.

[58] Kumar S, Mukherjee S, Singh RK, Chatterjee S, Ghosh AK (2011). Structural and optical properties of sol-gel derived nanocrystalline Fe-doped ZnO. J Appl Phys.

[59] Goodenough JB (1963) Magnetism and the chemical bond. Interscience, John Wiley And Sons, p 165.

[60] Janisch R, Gopal P, Spaldin NA (2005). Transition metal-doped TiO2 and ZnO-present status of the field. J Phys Condens Matter.

[61] Nakamura S, Soeya S, Ikeda N, Tanaka M (1993). Spin-glass behavior in amorphous BiFeO3. J Appl Phys.

[62] Vincent E, Dupuis V, Alba M, Hammann M, Bouchaud JP (2000). Aging phenomena in spin-glass and ferromagnetic phases: Domain growth and wall dynamics. Europhys Lett.

[63] Chang H, Guo YQ, Liang JK, Rao GH (2004). Magnetic ordering and irreversible magnetization between ZFC and FC states in RCo5Ga7 compounds. Magn J Magn Matter.

[64] Xing GZ, Yi JB, Wang DD, Liao L, Yu T, Shen ZX, Huan CHA, Sum TC, Ding J, Wu T (2009). Strong correlation between ferromagnetism and oxygen deficiency in Cr-doped In _2_O_3−*δ*_ nanostructures. Phys Rev B.

[65] Chu DW, Zeng YP, Jiang DL (2007). Synthesis and growth mechanism of Cr-doped ZnO single-crystalline nanowires. Solid State Commun.

